# Metastatic Prostate Adenocarcinoma Presenting as Acute Quadriplegia: A Case Report and Racial Disparity Analysis

**DOI:** 10.7759/cureus.27646

**Published:** 2022-08-03

**Authors:** Nadia G Obaed, Maria Silberstein, Miriam Zylberglait

**Affiliations:** 1 Allopathic Medicine, Nova Southeastern University Dr. Kiran C. Patel College of Allopathic Medicine, Fort Lauderdale, USA; 2 Internal Medicine, Aventura Hospital and Medical Center, Aventura, USA; 3 Internal Medicine/Geriatrics, Aventura Hospital and Medical Center, Aventura, USA

**Keywords:** social determinants of health, prostate screening, racial disparity, cancer epidemiology, metastatic spinal cord compression, metastatic prostate carcinoma

## Abstract

Prostate cancer is the most frequently diagnosed malignancy in males with the highest incidence and mortality among African Americans. Most prostate cancers are low-grade and slowly progressive. ^ ^Prostate cancer can be asymptomatic in the early stages, as exemplified by diagnosis through incidental findings, but typically manifests as a change in urinary habits and characteristics, including frequency and dysuria. If diagnosed at the time of distant metastases, then the patient may complain of bone pain in the hips, legs, or feet, or lower extremity edema.

We present the case of a 74-year-old African American male with no past medical history who presented to the emergency department with acute quadriplegia secondary to metastatic spinal cord compression. The patient required admission to the intensive care unit (ICU) and his quadriplegia was successfully treated with cervical arthrodesis, laminectomy, spinal instrumentation, and fusion, high-dose intravenous (IV) steroids, and physical and occupational therapy.

Overall, the purpose of this case report is to critically review and investigate the factors behind a patient's atypical, rare, and underreported initial presentation of metastatic prostate cancer. The study discusses the literature on advancements in prostate cancer screening and highlights the importance of a broad differential. Most remarkably, the vignette prompts an analysis of the racial disparity gap in prostate cancer diagnosis and treatment demonstrates the need for further research toward improved health outcomes, and proposes multiple avenues to promote health equity.

## Introduction

Background and epidemiology

The prostate is a small retroperitoneal reproductive gland in males surrounding the prostatic urethra. Anatomically, the prostatic parenchyma is divided into four distinct zones- peripheral, central, transitional, and periurethral, or five lobes- anterior, posterior, medial, and two lateral lobes. Carcinomas of the prostate generally form in the peripheral zone of the posterior lobe where the prostatic ducts empty into the urethra, thus contributing to urinary reflux [1,2]. Prostate cancer is the most frequently diagnosed malignancy in males accounting for 26% of new cancer cases with an incidence of 248,530 in 2021 [3]. Regions including the Caribbean, South Africa, and Lithuania bear some of the highest mortality rates, with prostate cancer being the second leading cause of cancer death in men worldwide [4]. According to the NIH SEER (National Institute of Health Surveillance, Epidemiology, and End Results) Program, there were 74% of localized and 7% of distant-stage (defined as metastasis) new prostate cancer diagnoses in the 2011-2017 period [3]. Furthermore, the percentage of deaths is higher in African Americans and men aged 75-85 years in the United States alone [3]. The mortality of prostate cancer has decreased by more than half in the past thirty years with an average mortality rate of 19 per 100,000 men between 2014-2018 [3]. Whether the decline in mortality derives from increased awareness, frequency of Prostate-Specific Antigen (PSA) screenings, or PSA-driven prostatectomy is still debatable.

Clinical presentation

Prostate cancer can be asymptomatic in the early stages, as exemplified by cases of random discovery through incidental findings. Although, prostate cancer typically manifests as a change in urinary habits including difficulty voiding, dysuria, frequency, nocturia, visible hematuria, and post-micturition dribble [3,5]. Erectile dysfunction, despite being common in the aging male population, has also shown an association with prostate cancer [6]. However, if prostate cancer is diagnosed at the time of distant metastasis, then the patient may complain of bone pain in their hips, legs, or feet, or lower extremity edema [5]. Hematogenously spread prostate cancer cells have demonstrated a strong tropism for bone due to a favorable microenvironment. The abundant red marrow within the axial skeleton provides malignant cells with the proper seeding ground to induce osteolytic, osteoblastic, and mixed bony lesions [7]. 

Diagnosis and prognosis

An asymmetric, hard, irregular mass palpated on a digital rectal exam (DRE) could be indicative of prostate cancer. A meta-analysis determined that DRE had a low sensitivity (51%) and specificity (59%) for detecting prostate cancer, however, the study had high levels of inter-group heterogeneity [8]. The PSA screening test has been in use for decades with a sensitivity of 21% and specificity of 91% for prostate cancer screening, but more accurate and newer tests including the prostate health index (PHI) and *PCA3* may become the recommended initial screening test of choice [9]. Most prostate cancers are low-grade and slowly progressive. The staging and grade of prostate cancer help determine the extent of the disease, applicable treatment options, and the five-year survival estimate of patients. Clinical staging consists of a DRE, PSA levels, and a Gleason score, while pathologic staging is based on prostate tissue or lymph node biopsy [2]. Based on a study using SEER data, localized and regional prostate cancer (stage I to IIIC) have a relative five-year survival of 100% at the time of diagnosis versus 30.6% with metastasis (stage IV) [3].

The purpose of this study is to report an atypical, rare, and underreported initial presentation of metastatic prostate adenocarcinoma, review the literature on the early detection and diagnosis, and analyze the skewed incidence of prostate cancer with a focus on closing the racial disparity gap and promoting health equity.

## Case presentation

A 74-year-old African American male with no reported past medical history presented to the emergency department (ED) as a stroke alert with acute bilateral upper and lower extremity weakness after a ground-level fall. The patient complained of mild straining to urinate, intermittent dyspnea, generalized weakness, and bilateral lower extremity swelling, which he noticed within the past couple of weeks. He denied any fall or trauma prior to symptom onset, history of similar episodes, and prior neck or back pain. The patient also denied a history of cancer. He stated that he had not seen a doctor in over twenty years. 

On his physical exam, the patient had 2/5 strength throughout his bilateral upper and lower extremities. Neurologic examination showed no bulbar involvement, but a severely dull sensation throughout, with the patient stating that the pinprick felt like ice.

**Figure 1:**
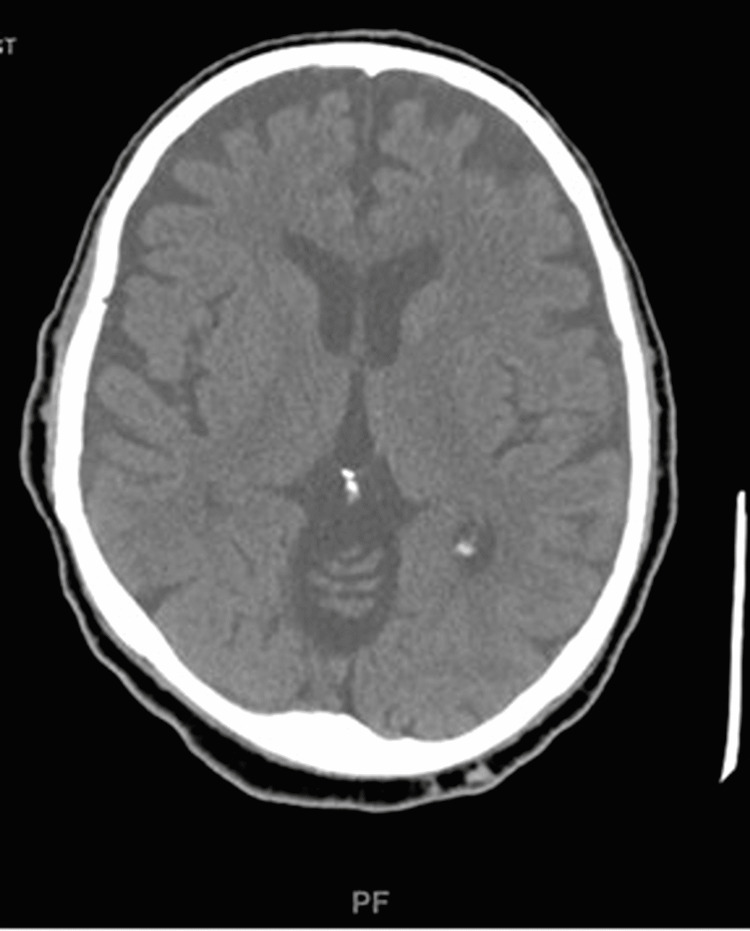
CT Brain without contrast. CT of the brain was performed without intravenous (IV) contrast showing small bilateral frontal crescentic shaped mixed density subdural hematomas measuring up to 7 mm on the left and 5 mm on the right in maximal thickness and no evidence of ischemic infarct.

**Figure 2:**
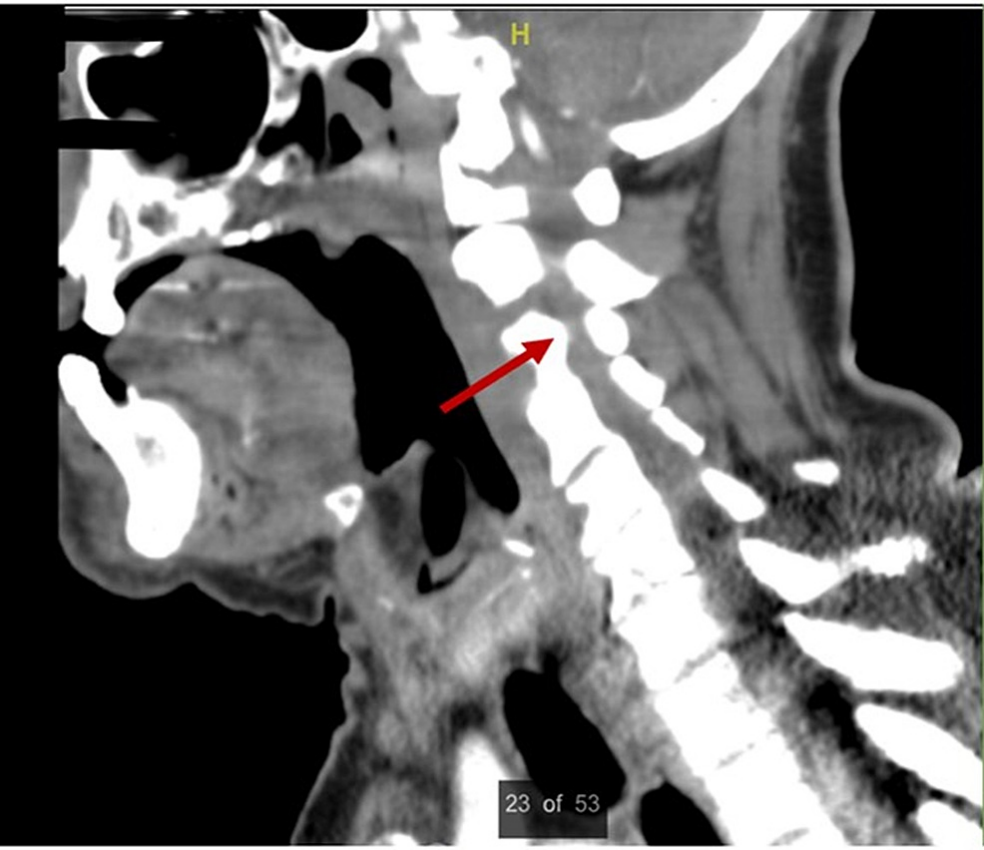
CT scan of the neck and spine. CT scan showing C3 cord compression, prominent diffuse sclerotic and lytic appearing osseous structures, and multilevel degenerative cervical spine changes.

**Figure 3:**
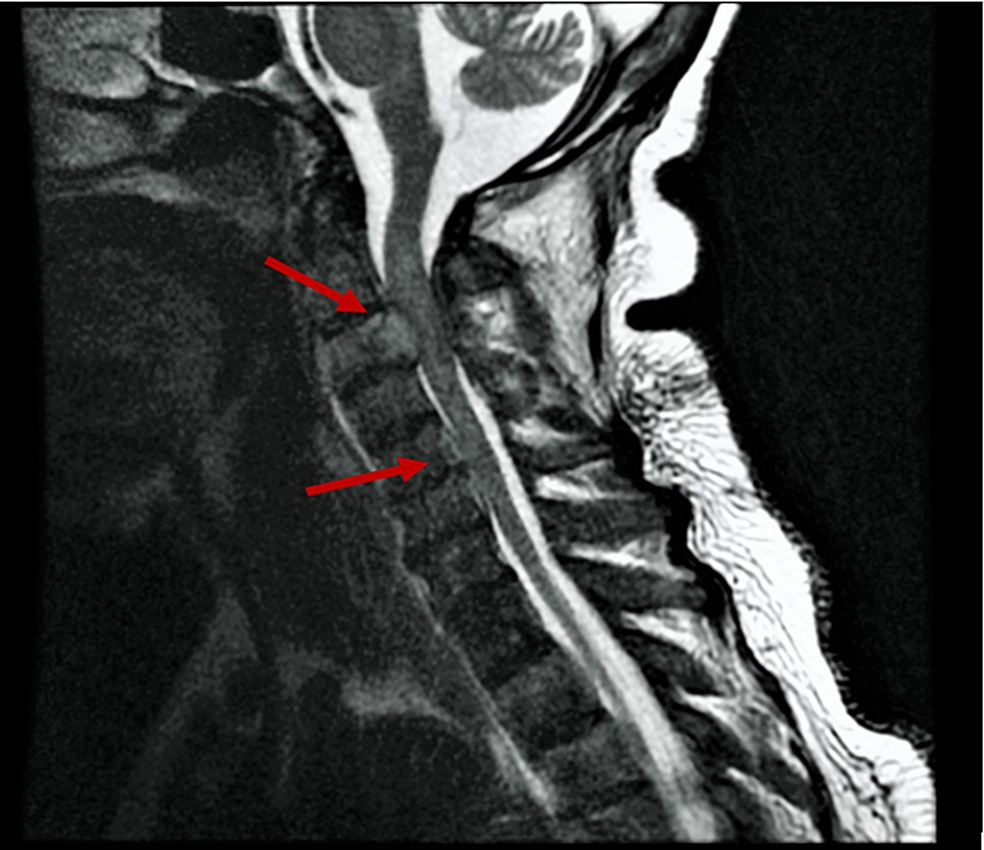
MRI of the spine. Spine MRI showing C3 lesion with posterior extension to the ventral and lateral epidural spaces encircling the spinal cord associated with cord compression, edema, and expansion (top arrow). There is an extensive osseous metastatic disease with cortical breakthrough along with compression from the C5-C6 vertebral bodies that effaces, but does not surpass the thecal sac at the C5-C6 level (bottom arrow).

The initial differential diagnosis was vertebrobasilar stroke due to the acuity of the patient’s weakness. Guillain Barre syndrome was also considered. Per the basis of an acute stroke protocol, the patient immediately received an initial non-contrast CT scan of the brain which was only remarkable for small bilateral frontal subdural hematomas (Figure 1). Further CT scans of the brain and neck corroborated the first CT's results and additionally demonstrated bilateral stenosis of the V2 arteries, anterior cervical epidural fluid collection, C3 cord compression, and mixed lytic and sclerotic osseous structures (Figure 2). MRI of the spine was done to further characterize the C3 lesion (Figure 3).

Upon initial laboratory assessment, the patient was found to have an elevated alkaline phosphatase level (636 IU/L). The patient’s total PSA and free PSA were high at 3314 ng/mL and 588 ng/mL, respectively. Cardiovascular and ischemic pathology was evidenced with elevated troponin, NT-proBNP (N-terminal-pro hormone B-type natriuretic peptide), and lactic acid. A complete hemogram was significant for decreased hemoglobin at 11.8 gm/dL. His metabolic panel was within normal limits. Liver function tests showed increased liver enzymes (AST {aspartate aminotransferase} at 347 U/L and ALT {alanine aminotransferase} at 182 U/L). 

Given the patient's age, ethnicity, urinary symptom, and PSA levels, prostate adenocarcinoma was a top differential. Another strongly considered differential was multiple myeloma due to preceding weakness, anemia, and osteolytic lesions within the vertebral column. Other possible differentials prior to this patient’s workup included gastrointestinal (GI) malignancy, spinal tumor, nontraumatic cervical disc rupture, and stroke. 

Focused workup

**Figure 4:**
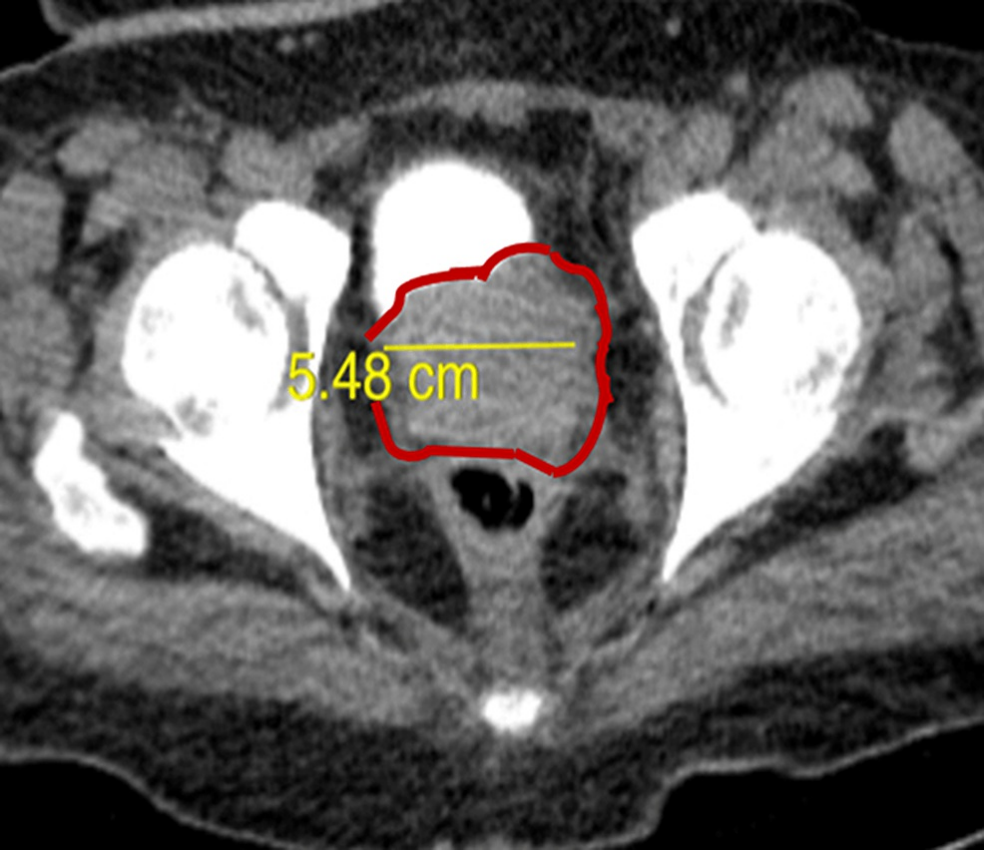
Axial CT scan of the pelvis. Pelvic CT scan showing enlarged prostate measuring 5.48 cm in diameter with indentation along the posterior aspect of the bladder.

**Figure 5:**
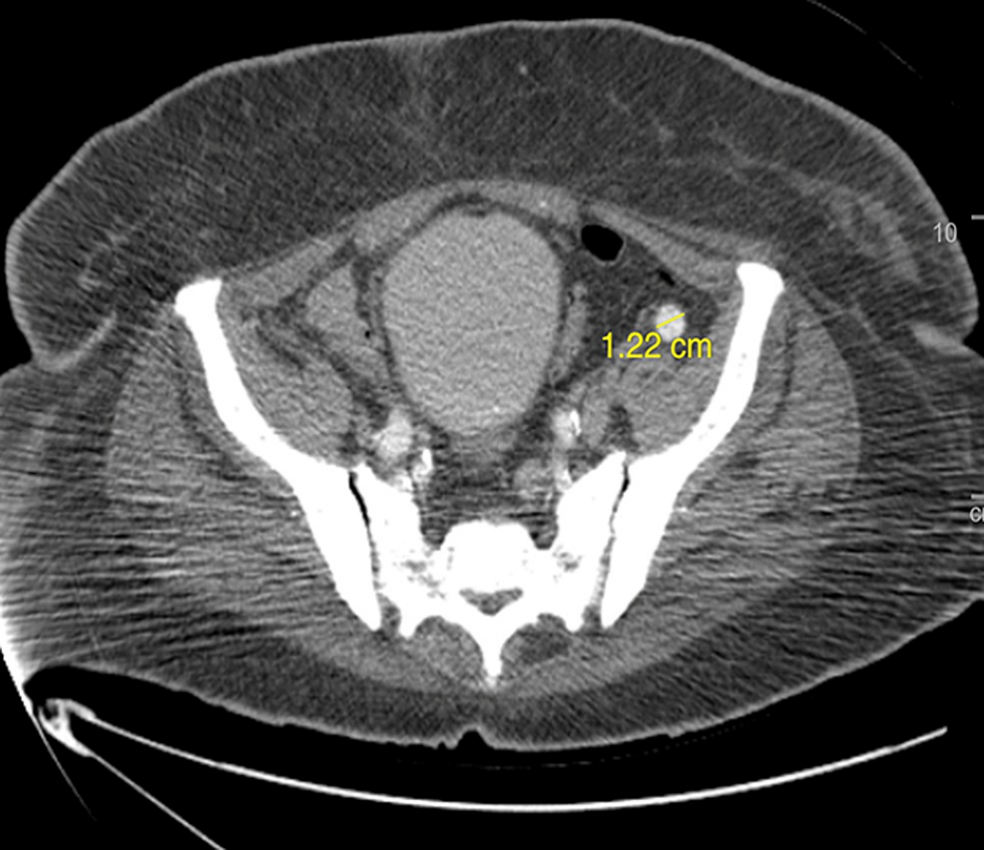
Axial CT scan of the pelvis. Axial CT scan showing enlarged right external iliac lymph node measuring 1.22 cm in diameter.

Serum protein electrophoresis (SPEP) was conducted as multiple myeloma was part of the differential. The SPEP displayed decreased gamma globulin and albumin, but elevated beta-2-microglobulin and alpha-1 globulin. A CT scan of the chest, abdomen, and pelvis with contrast showed lung micronodules, diffuse skeletal metastases, enlarged external iliac lymph nodes, aneurysm of the right common iliac artery, and aortic arch (Figure 4). Upon imaging, the prostate was enlarged with indentation along the posterior aspect of the bladder (Figure 5).

High clinical suspicion of prostate carcinoma remained due to significantly elevated PSA, enlarged prostate, symptoms of urinary retention, and mixed osteoblastic and osteolytic vertebral lesions that were concerning for a metastatic disease process.

Therapeutic intervention and results

Upon the patient's initial presentation to the ED, a hard cervical collar was placed. He was started on Decadron 3 mg intravenous (IV) (Fera Pharmaceuticals, Locust Valley, NY) every eight hours to relieve associated pain from inflammation and edema. Neurosurgery was indicated secondary to his large C3 expansile mass with symptomatic spinal cord compression. Posterior cervical arthrodesis (C2-C4), cervical laminectomies (C2-C3, partial C4), and posterior spinal instrumentation and fusion (C2-C4) were performed within two days of admission. 

The patient had improved motor strength equally to 4/5 in all four extremities. No postoperative or new focal neurologic deficits were found. The surgical pathology report noted that the C3 laminectomy fragments were positive for malignancy consistent with metastatic prostate carcinoma. More specifically, tumor cells were positive for pankeratin and NKX3. Monoclonal immunoglobulin was not detected by immunofixation. The patient was diagnosed with acute quadriplegia due to C3 spinal cord compression secondary to metastatic prostate malignancy. 

The patient was then transitioned to inpatient rehabilitation for ongoing physical therapy and strength exercise, directed to close outpatient follow-up with neurosurgery, hematology oncology, radiation oncology, and urology. The cervical collar was to remain on for the next four to six weeks until he was seen by neurology in outpatient follow-up.

## Discussion

The sudden onset of quadriparesis as the initial manifestation of prostate cancer in a male above the age of 50 years is rare and unreported. However, a couple of relevant studies do exist reporting progressive paresis or spinal cord compression secondary to vertebral fracture or cancerous mass growth from metastatic prostate cancer [10,11]. An acute presentation of spinal cord compression can be indicative of spinal tumor, infection, or trauma [12]. In elderly male patients with a history of limited to no primary care contact, a source of malignancy should be highly considered for investigation. The most common malignancies to metastasize to the spine include lung, breast, prostate, melanoma, kidney, and thyroid cancers [12]. There is an even wider differential that should be investigated when a lesion is found in the bone and can be split based on the presence of lytic, blastic, or mixed processes. 

Metastatic cancer to the spinal cord occurs in 5-10% of cancer patients, and 10% of patients presenting with new spinal metastasis were previously unaware of their cancer [13]. The lumbar region of the spine is most commonly involved in symptomatic spinal metastasis (70%), while cervical involvement accounts for only 10% of cases [13]. Comparative studies regarding outcomes after surgery for the different spinal cord regions affected in this oncologic emergency are limited. One study showed that the outcome of metastatic-related cervical spinal stenosis post decompressive and spine stabilization surgery had less improvement or maintenance of motor function as well as the highest deterioration in motor function compared to patients with post-surgery for thoracic or lumbar spinal metastasis [14]. 

The clinical relevance of metastatic bone disease (MBD) reaches beyond an individual’s health outcome such that MBD accounts for approximately $12.6 billion of the $74 billion national oncologic expenditure [15]. Given the annual rise in healthcare costs, the burden is speculated to be drastically higher today. Spinal cord compression, when classified as a skeletal-related event secondary to MBD, is associated with higher average management costs (~$54,444), longer inpatient stays, increased hospitalizations, and more procedures per patient [15]. Although the prevalence of MBD should actually decrease with the expansion of improved early detection screenings and therapies, this does not fully address one of the problems presented in the report-why do African American males have the highest predominance of prostate cancer? Our call through this report is to recognize the paths that need to be taken to decrease this racial health disparity. The health disparity can be imagined as a cycle with detection, incidence imbalance, lack of racial concordance, bias, and research inclusion as the wheels driving the inequity. 

The relative incidence of bone metastasis in patients with prostate cancer is greater than 60% [3]. To prevent cancer from getting to such an aggressive stage, prostate cancer screening and combined biopsy strategies for diagnosis are vital. Combining biopsy strategies allows doctors to find up to 33% more prostate cancers according to Dr. Leonard Marks through the fusion of suspicious lesions on MRI with the real-time US to guide needle biopsies [16]. In terms of screening the most high-risk population, the epidemiology of prostate cancer should be understood. The high-risk population is African Americans in part because of lifestyle, diet, and largely genetics [17]. The cumulative androgen exposure stemming from diet acting on the hormonal pathway to increase testosterone, in utero exposure affecting the gonadostat feedback loop, and sustained increase with age correlates to the 70% higher rate of prostate cancer incidence in African Americans [18].

Societal factors and access to care have the largest influence on the skewed incidence, overpowering the aforementioned factors. This factor also contributes to the 2.5 times higher mortality rate among African Americans [19]. This population receives fewer PSA screenings, so they are most likely diagnosed with a later stage of prostate cancer [20]. A later stage makes prostate cancer more difficult to treat and is associated with an increased risk of relapse. At the start of the COVID-19 pandemic, there had also been a shift in cancer resource allocation which consequently made it less likely for black men to receive prostatectomy [21]. Furthermore, they are less likely to have health insurance, access to high-quality care, and more likely to have a lower socioeconomic status [19].

The most prevalent treatment options consist of prostatectomy and radiation therapy [10]. Selecting the most appropriate treatment for a patient largely depends not only on stage, but also on disease progression, patient’s overall health status, and own goals of care. A large, randomized trial was conducted in which participants had specific treatment regimens and regular follow-up, even up to ten years, and the results suggested that it was external factors instead of genetics impacting the prostate cancer rate differences among races [19]. The study found that black men did not have an increased risk of dying from prostate cancer compared to white men with a similar stage of the disease, but black men do die more often from other causes like cardiovascular disease [19]. Therefore, the implied disparity is access to quality care. The trial even showed that black men had higher cure rates than white men who were both treated with radiation at the same stage of the disease [19].

Another problem underlying the racial disparity revolves around the role of healthcare professionals in screening. The AUA 2019 data showed that black urologists were only 2% of the workforce, they also only make up 5% of all physicians and 5% of urology residents [22]. Ethnic and racial concordance is not present in most urologist-patient interactions. The discordance couples with a significant blanket mistrust of the healthcare system and limits patient satisfaction [23]. Thus, discordance can also factor into the late presentation time of prostate cancer diagnosis in African Americans. However, increasing cultural competence, for instance through educational training programs and patient navigators, promotes greater participation in shared decision-making, treatment adherence, and health literacy [23]. Efforts must also be made to support diversity in educational programs and healthcare careers. 

A study showed that non-black physicians with higher levels of implicit bias were shown to talk more during consultations with black patients [23]. The study result is reflective of the diminished satisfaction black patients feel when listened to less than others [23]. Strategies employed to decrease implicit bias (individuation, partnership building) in both the current and new generations of physicians will improve equality in health outcomes. Learning how to empower patients to take an active and equal role in their health, as well as educating them to decrease any knowledge gap enhances their overall care. 

The National Cancer Institute has a Prostate, Lung, Colorectal, and Ovarian (PLCO) Cancer Screening Trial in which only 4% of participants were black. For data to accurately reflect clinical health practices for African Americans, their participation in research must increase [24]. As research continues to accumulate on the best approach for screening the general population, primary care physicians may want to incorporate standard screening for all new African American males above the age of 40 years with re-screening intervals depending on the patient’s PSA level and general health. This earlier and standardized screening would be indicated based on their greater risk to benefit ratio.

## Conclusions

In summary, our case reviews a very rare, but possible event in which an elderly African American male without adequate access to health care presented with acute onset quadriplegia secondary to spinal cord injury from metastatic disease discovered on admission. Not only does this report highlight the necessity of a broad differential, but it most remarkably demonstrates that efforts must be taken to close the gap in racial health disparities perpetuating worse health outcomes. 
